# Examining working and episodic memory in young adults with anhedonia

**DOI:** 10.3758/s13415-025-01315-y

**Published:** 2025-06-02

**Authors:** Sofia Uribe, Holly J. Bowen, Alicia E. Meuret

**Affiliations:** Department of Psychology, Southern Methodist University, Dallas, TX USA

## Abstract

Depression is associated with impairments in memory processes. Evidence suggests that poorer recognition of positive information and quicker disengagement with positive information from working memory in depressed versus nondepressed individuals. The working memory deficits are speculated to be downstream effects of a taxed working memory due to rumination processes that impair reward learning leading to anhedonia symptoms. Downstream effects are also hypothesized for episodic memory with impairments particularly for positive information due to dopamine dysregulation from anhedonia affecting memory formation processes. To examine the association of memory impairments in individuals with mild-to-severe anhedonia and depressive symptoms, 108 young adults completed a working memory task where they had to remember an abstract shape while presented with two consecutive positive, neutral, or negative images. This task was followed by a surprise episodic memory recognition test for the images the next day. The Drift Diffusion Model index *drift rate* was used to examine whether anhedonia severity predicted evidence accumulation rates during working and episodic memory retrieval. Contrary to expectations, based on multivariate models, anhedonia severity did not predict evidence accumulation rate for any specific valence in either task. These results suggest that anhedonia symptoms may not be uniquely associated with memory differences for emotionally valenced compared with neutral stimuli. Further studies should investigate the role of specific facets of anhedonia, including anticipatory reward and use different paradigms and neurophysiological measures, to examine the proposed hypotheses.

**Supplementary Information:**

The online version contains supplementary material available at 10.3758/s13415-025-01315-y.

## Introduction

Depression is commonly associated with cognitive symptoms, such as deficits in memory, executive function, attention, and decision-making (Fava et al., 2006; Lee et al., 2012; Trivedi & Greer, 2014; see LeMoult & Gotlib, 2019, for a review). The impairments in long-term memory include the preferential recall of negative over positive information compared with nondepressed participants who demonstrate enhanced memory for positive information (Gotlib & Joormann, 2010; James et al., 2021; Marchetti et al., 2018). Previous findings indicate that memory impairments may also occur in working memory—the temporary storing and manipulation of information for an immediate goal (Baddeley, 2000). Depressed individuals show greater difficulties removing irrelevant negative stimuli from working memory (Joormann & Gotlib, 2008) and disengage faster from positive information (Levens & Gotlib, 2010) compared with nondepressed. The memory deficits in depression may be maintaining depressive mood by altering the effectiveness of other emotion regulation strategies (e.g., mood-incongruent recall; Gotlib & Joormann, 2010; Rusting & DeHart, 2000).

Anhedonia is a core symptom of depression and is described by markedly diminished interest or pleasure in things that used to previously provide it (DSM-V; American Psychiatric Association, 2013). Anhedonia is characterized by dysfunction in reward processes, including reward learning, and low positive affect (Craske et al., 2024; De Fruyt et al., 2020). One proposed hypothesis suggests that rumination, the repetitive and passive pattern of focusing on depressive symptoms (Nolen-Hoeksema, 1991), is indirectly associated with anhedonia through working memory (Rutherford et al., 2023). Rumination taxes working-memory abilities, and the taxed working-memory abilities then disrupt an individual’s ability to learn from rewarding cues in the environment, because they are not able to hold updated, new positive information, and consequently, contribute to anhedonia (Collins & Frank, 2018; Craske et al., 2024; Frey et al., 2023; Rutherford et al., 2023). That is, working memory deficits in updating and maintaining positive information that is not congruent with ruminative thinking may contribute to and exacerbate anhedonia symptoms. Given the role of memory impairments in maintaining depressive symptoms, it is imperative to understand the mechanisms of the pertinent memory processes. However, the link between working memory processes and anhedonia remains unexplored.

Compared with working memory, episodic memory processes in depression have been examined more thoroughly. Guided by cognitive theories (Beck, 1967), research on episodic memory processes in depression largely focuses on memories of negative stimuli and their association with depression (Marchetti et al., 2018). The mechanisms underlying poor recognition for positive information remain largely inconclusive. Dillon (2015) proposed that the reward circuit dysfunction in depression is associated with stress on the mesolimbic dopamine pathway and, thus, disrupts reward signals (i.e., positive prediction errors, PPEs) that trigger consolidation to create a memory for positive/rewarding events. In a study by Dillon et al. (2014), adults with depression had a reduced neural response to reward stimuli in the dopaminergic midbrain and parahippocampus compared with healthy adults. The reduced neural response was associated with impaired memory for the rewarded stimuli, which supports the proposed hypothesis. Limitations of that study included a very short retention interval (i.e., testing directly after encoding), differences in reaction time were not examined, and the recognition test was expected by the participants (i.e., participants were told they will be tested after the encoding task), which may influence the encoding strategies they used. Furthermore, while they found significant group differences on depression and consummatory pleasure scales (i.e., Beck Depression Inventory-II, Beck et al., 1996; and Snaith-Hamilton Pleasurer Scale, Snaith et al., 1995), they did not examine to what extent the impaired memory for rewarded stimuli is associated with symptoms of anhedonia. Understanding whether low positive mood and anhedonia, compared with negative depressive symptoms, such as low mood or hopelessness, is associated with the poor or slower recognition of positive information holds the promise for more targeted interventions by illuminating the mechanisms of memory impairments in depression.

**Fig. 1:**
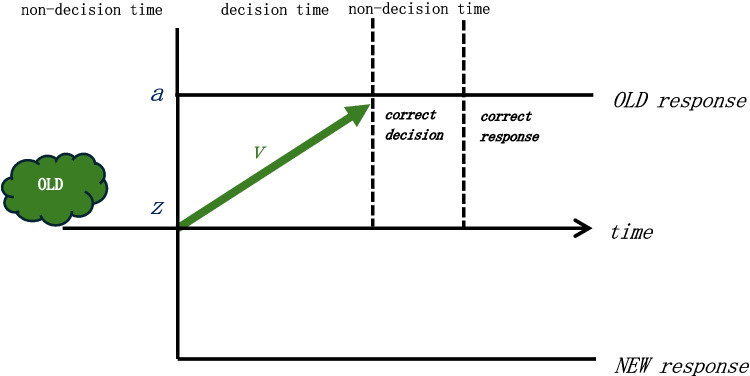
Drift diffusion model. The schematic of the DDM for an image that is “old” and a correct response (e.g., hit) includes 1) the nondecisional processes, reported as nondecision time, include stimulus perception and motor responses; 2) the decisional process captures the move from a starting point to either boundary representing the decision; 3) the boundary separation (parameter *a*) represents the distance between the two boundaries and is an indication of how much information is required to reach the decision; and 4) the parameter of interest for the current study was the drift rate (*v*), or the average rate at which the memory of the stimuli accumulates towards either the upper (“old”) or lower boundary (“new”). A choice is made when a boundary is reached. Higher drift rates are associated with quicker reaction times and more accurate responding

Most paradigms used to examine memory performance in depression consist of two-choice recognition memory tasks where participants are asked to identify whether they have seen the stimulus before (i.e., select “old”) or if it is a stimulus they have not seen before (i.e., select “new”). Because performance, based on accuracy (e.g., percentage correct, hits) and reaction time, is measured separately, information about the latent cognitive processes is missed (e.g., response caution, speed of evidence accumulation processing). Computational models, such as the Drift Diffusion Model (DDM; Ratcliff & McKoon, 2008), are sequential sampling models used to study the underlying cognitive processes from overt behavioral data, thus capturing more robust differences that observable behavioral data (e.g., accuracy and reaction time) may not reveal (Myers et al., 2022). The DDM integrates reaction time and accuracy in a two-choice decision: it captures the time it takes (i.e., decision time) to accumulate evidence to reach a decision (i.e., boundary) when a stimulus is presented (i.e., starting point). There are two boundaries representing the two decision recognition choices: an upper boundary (“old”) and a lower boundary (“new”). There are also nondecisional processes, reported as nondecision time (parameter t0), which include stimulus perception and motor responses. The decisional process captures the move from a starting point (parameter *z*) to either boundary representing the decision. The boundary separation (parameter *a*) represents the distance between the two boundaries and is an indication of how much information is required to reach the decision. The parameter of interest for the current study was the drift rate (*v*) or the average rate at which the memory of the stimuli accumulates towards either the upper (“old”) or lower boundary (“new”). A choice is made when a boundary is reached. Higher drift rates are associated with quicker reaction times and more accurate responding. See Fig. 1 for a visual representation.

In a memory retrieval task, the drift rate captures the strength of the memory. That is, the speed in which evidence is accumulated depends on how well the test stimulus matches its representation in memory (Ratcliff, 1978). When the test stimulus is well-encoded, the match with its representation is good, so the evidence accumulates efficiently toward the “old” boundary. When the stimulus is new, there is no evidence of a match, so the evidence accumulates efficiently toward the “new” boundary. When the stimulus is poorly encoded, the match is weak so the evidence accumulates more slowly and may end at the incorrect “new” boundary. In this case, the recognition accuracy would decrease. By using this model, we can examine whether evidence accumulation is higher or lower for specific stimuli in anhedonia, which can be instrumental in understanding what is driving the memory impairments in depression.

Three studies have utilized computational models to examine the association between episodic memory processes and negative symptoms of depression. White et al. (2009) found that while there was no difference between dysphoric and nondysphoric college students in accuracy or reaction time when these outcomes were analyzed separately for an emotional memory recognition task, there was a significant difference in a DDM-index drift rate. That is, the dysphoric group had a lower drift rate for positive words, indicating a lower accumulation of evidence during the retrieval of positive memories. More recently, Maksmimovskiy et al. (2022) compared behavioral data from a surprise recognition memory test for stimuli from an oddball paradigm (i.e., participant must respond to stimulus types presented less frequently) between participants with low depressive symptoms (i.e., low depression group) to more severely depressed adults (i.e., high depression group). The high depression group had lower drift rates for old positive pictures; that is, memory evidence accumulated more slowly and less accurately for positive pictures compared to the low depression group. Similarly, but to a lesser extent, the drift rates were lower for the old neutral pictures in the high depression group, whereas the drift rates were similar between groups for the old negative pictures. Finally, Cataldo et al. (2023) also examined behavioral data from multiple memory tests, including the drift rate from a recognition memory task, and similarly found that as depressive symptoms increased, the drift rate for old (i.e., studied) positive words decreased. Overall, they found that as depressive symptoms increased, participants demonstrated higher drift rates towards an “old” response for negative words and higher drift rates towards a “new” response for positive words, regardless of whether the images were “old” or “new.” Taken together, the three studies suggested that depressive symptoms are associated with differential drift rates based on the valence, in that depressive symptoms were associated with lower drift rate of positive “old” images in all three studies.

These studies offered novel insights into episodic memory deficits as they associate to depressive symptoms; yet the extent to which anhedonia symptoms relate to the differences in drift rates for positive stimuli remain unexplored. Furthermore, to our knowledge, no previous research has examined evidence accumulation during working memory in adults with anhedonia. Lastly, based on behavioral and neuroimaging findings working and episodic memory are not entirely parallel systems but interactive (Hoskin et al., 2019). Previous findings suggest that different cognitive strategies during a working memory task can relate to performance in episodic memory in different ways. For example, reappraisal of a distractor may lead to successful coping with distraction during working memory (i.e., having no impairments in maintaining target stimuli) and to a more robust encoding (i.e., better episodic memory; Dillon et al., 2007; Dolcos et al., 2013; Iordan et al., 2013). Other strategies, such as suppressing, ignoring, or not paying attention to the distractors, may lead to successful working memory performance but worse episodic memory. However, whether anhedonia severity is associated with the ability to engage in different cognitive strategies during successful working memory trials remains unknown.

The present study examined evidence accumulation rates in a working memory task with emotionally-valenced distractors followed by an episodic memory task for the distractors. Of particular interest was whether evidence accumulation in these two tasks varied across participants with a range of mild-to-severe anhedonia symptoms. We hypothesized that as anhedonia severity increased 1) the average drift rate across all trials with negative distractors in the working memory task would decrease, indicating a decreased ability to remove negative distractor images from working memory; 2) the average drift rate across all trials with positive distractors in the working memory task would increase, indicating an increased ability to remove positive distractors images from working memory; and 3) the drift for positive target images in the episodic memory task (i.e., positive distractors presented, and incidentally encoded, in working memory task) would decrease, indicating a lower evidence accumulation of positive stimuli. Lastly, 4) we had no a priori hypothesis on whether episodic memory performance after a successful working memory trial would differ as anhedonia severity increased.

## Methods

### Participants

Participants (*n* = 108) were college students and volunteers from the greater Dallas community. The target sample size was determined by reference to a similar prior study (Weintraub-Brevda & Chua, 2019), which was sufficiently powered (95%) to detect a medium-sized effect with the same behavioral task (*n* = 30 per group). Given an additional analysis we aimed to conduct, the target size was increased to 100 (*n* = 50 per group). G*Power 3.1 (Faul et al., 2009) was used to determine the sample size for 80% power to detect a medium-sized effect for a linear multiple regression with six predictors. Students received research credits and participants recruited from the community received a gift card with a small monetary compensation for participating.

To ascertain a range of depressive and anhedonia symptom severity, the high anhedonia sample had to endorse both anhedonia and at least mild depression during the self-report screening questionnaires completed via REDCap. To meet criteria, the high anhedonia sample had to 1) endorse the second diagnostic criteria of Major Depressive Disorder (MDD): *Marked diminished interest/pleasure in all (or almost all) activities most of the day, nearly every day* (Diagnostic and Statistical Manual of Mental Disorders 5 th ed.; DSM-5; American Psychiatric Association, 2013) by responding “yes” to the criteria as part of the self-report screener, 2) meet clinical anhedonia levels defined as a score greater than 2 on the Snaith-Hamilton Pleasure Scale (SHAPS; Snaith et al., 1995), and 3) report at least mild level of depression defined as a score of 13 or greater on the Beck Depression Inventory-II (BDI-II; Beck et al., 1996). Screened participants were excluded if they met the depression criteria but did not meet the anhedonia criteria, or vice-versa. Participants in the control group without anhedonia or depressive symptoms had to respond “no” to the second diagnostic of MDD during the self-report screener, score a 2 or less on the SHAPS, and less than 13 on the BDI-II. We recruited equal number of participants from each group.

Additionally, individuals with psychiatric disorders or medical illnesses, which potentially interfere with memory performance, were excluded. This included a self-reported lifetime history of bipolar disorder, psychosis, organic brain damage, history of severe, uncontrolled medical illness or instability, and current substance abuse. Participants provided informed consent before participating, and the study was approved by the university’s institutional review board.

### Measures

To assess anhedonia symptoms severity, our primary predictor measure, the 17-item Dimensional Anhedonia Rating Scale (DARS; Rizvi et al., 2015) was utilized. Each item is rated on a five-point scale ranging from 0 (*not at all*) to 4 (*very much*). All items are summed for a total score that ranges from 0 to 68, with higher scores indicating less anhedonia. There are four domains—hobbies, social activities, food/drink, and sensory experiences, for which each participant provides their own examples. They subsequently answer standardized questions within each domain (e.g., *I would enjoy these activities; I would make an effort to get/make these foods/drinks*). A score per domain can be calculated by summing the items within each domain. The DARS has a high internal consistency of 0.91 to 0.96 for the total score and 0.75 to 0.92 for subscales across studies (Rizvi et al., 2015). Cronbach’s alpha for this study was 0.96.

*Depressive symptoms severity* was measured using the 21-item Beck Depression Inventory-II (BDI-II; Beck et al., 1996). All items are rated on a four-point scale from 0 to 3, with higher scores indicating greater severity. Each item is summed to a single score that ranges from 0 to 63. The BDI-II has a 1-week test–retest reliability of *r* = 0.93 and a Cronbach’s coefficient alpha of 0.91 for outpatients with depression (Beck et al., 1996). Cronbach’s alpha for this study was 0.95.

*Consummatory anhedonia* was assessed using the 14-item Snaith-Hamilton Pleasure Scale (SHAPS; Snaith et al., 1995). Each item is a confirmatory statement about an enjoyable situation typically encountered in daily life across cultures (e.g., social interactions, interests/hobbies, food/drink) rated using the following four-point scale: 0 (*strongly agree*); 1 (*agree*); 2 (*disagree*); or 3 (*strongly disagree*). A rating of zero or one counts as a zero and a rating of two or three is counted as a one. Therefore, all items are summed for a total score that ranges between 0 and 14, with higher scores indicating higher levels of state anhedonia. The SHAPS has a high internal consistency based on Cronbach’s alpha of 0.91 in a healthy and depression sample (Franken et al., 2007; Nakonezny et al., 2010). Cronbach’s alpha for this study was also 0.91.

### Behavioral Task Stimuli

The image stimuli used for the behavioral tasks were chosen from the International Affective Picture System (IAPS; Lang, Bradley, & Cuthbert, 1997). There were 90 positive, 90 negative, and 90 neutral images selected. Average valence category valence ratings were calculated using the normed data (*M*_*rating*_ positive = 6.86 [*SD* = 0.47], *M*_*rating*_ neutral = 4.8 [*SD* = 0.57], *M*_*rating*_ negative = 2.74 [*SD* = 0.61]). A one-way analysis of variance (ANOVA) yielded a significant difference in the normed valence ratings based on valence categories, *F*(2, 267) = 1248.86,* p* < 0.001, η_p_^2^ = 0.9, between the three categories. That is, the positive images were rated as more pleasant than the neutral, *p* < 0.001, and the negative images, *p* < 0.001; and the neutral images were rated as more pleasant than the negative images, *p* < 0.001. Average valence category arousal ratings were calculated using the normed data (*M*_*rating*_ positive = 4.8 [*SD* = 0.93], *M*_*rating*_ neutral = 4.03 [*SD* = 0.95], *M*_*rating*_ negative = 5.51 [*SD* = 0.79]). Although all averages were within the mid-arousal range (3.66–6.32), a one-way ANOVA yielded a significant valence effect on the arousal ratings for the normed data, *F*(2, 267) = 61.52,* p* < 0.001, η_p_^2^ = 0.32, between the three categories. That is, the negative images were rated as more exciting than the neutral, *p* < 0.001, and the positive images, *p* < 0.001; and the positive images were rated as more exciting than the neutral images, *p* < 0.001.

The shape stimuli for the working memory task were line drawing abstract shapes from the Slotnick database (Slotnick & Schacter, 2004). The 180 black and white shapes were all prototypes to maintain uniqueness.

### Procedures

Participants who expressed interest in participating in the study were sent a set of screening questionnaires via REDCap to assess eligibility. The screening questionnaires assessed anhedonia endorsement using criteria two of the MDD diagnostic criteria, anhedonia level using the SHAPS, and current level of depressive symptoms using the BDI-II. Participants who were eligible based on the self-report questionnaires were invited to enroll in the study. All procedures were preregistered.

The study consisted of two virtual study visits over end-to-end encryption Zoom, 24 h to 36 h apart (*M* = 26.0 h, *SD* = 3.42 h). The participant was able to log in using a laptop (i.e., the task would not work if they tried to use a cellphone), and the behavioral tasks were standardized so that the images presented were the same size across computer monitors. Participants were encouraged to log in from a private, undisturbed location and were instructed to remove any distractors (e.g., email, music, cellphone). A study researcher remained on the zoom videocall during the entire duration of the study visits to answer any questions regarding the self-report measures and provide instructions for the behavioral tasks.

During the first visit, participants completed the informed consent, and a series of self-report measures, including the BDI-II, the DARS, and a demographics questionnaire. The self-report measures were completed through a unique link that was sent to the participant. Then, participants completed the working memory task. The first virtual visit took approximately 1 h, with an optional 5-min break between the self-report measure and the behavioral task. During the second virtual visit, the participant completed the episodic memory task followed by the image ratings task after the recognition test was completed. The second visit took approximately 45 min, including an optional 5-min break between tasks. Participants were then debriefed upon study completion.

### Behavioral paradigm

**Fig. 2:**
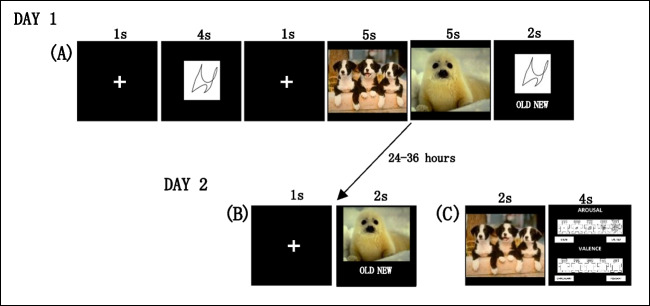
Behavioral tasks. (**A**) Working Memory Task; (**B**) Episodic Memory Task; (**C**) Picture Rating Task

Participants completed a two-part task adapted from Weintraub-Brevda and Chua (2019) to assess working memory abilities for abstract shapes with emotionally valenced distractors and episodic memory of those distractors. During part 1 (day 1), which assessed working memory, participants were instructed to keep abstract shape memoranda presented to them in mind, because they were going to be asked about them later. On some trials, one shape was presented (i.e., low load trial), and on some trials, two shapes were presented (i.e., high load trial). Participants were presented with a fixation cross for 1 s, shape memoranda for 4 s, another fixation cross for 1 s, followed by two consecutive positive, negative, or neutral images as distractors for 5 s each. The two distractors were of the same valence per trial. Participants were told to view the images but focus on remembering the shape memoranda. Then, a shape memorandum was presented for 2 s, and the participant had to judge whether the image was new (i.e., did not match the predistractor shape) or old (i.e., matched the predistractor shape). On high-load trials, participants were shown two shapes during the encoding phase. During the test phase, only one shape was presented, which could either be one of the two previously presented shapes (considered"old") or a completely new shape not shown before (Fig. 2). There was an equal number of trials (*n* = 30 each) for each distractor type (negative, positive, neutral), an equal number of working memory load (high load = two shapes; low load = one shape), and an equal number of old and new trials (*n* = 45 each). All trials were randomized.

During part 2 (day 2), participants completed the episodic memory recognition test. The test consisted of the 90 “distractor” images that were presented during the working memory task (i.e., target or old images for the recognition test) and 90 new images (i.e., lures). The recognition test was unexpected as participants were not told to remember the distractor images during the working memory task. During the episodic memory recognition test, participants were presented with a fixation cross for 1 s followed by an image for 2 s, which they had to judge as new (i.e., did not see the image the day before during the working memory task) or old (i.e., image was presented the day before during the working memory task) (Fig. 2). Trials were randomized, and there was an equal number of images across valences (negative, positive, neutral; *n* = 60 each) and an equal number of new and old images (*n* = 90 each).

Participants also completed 270 arousal and valence ratings of the negative (*n* = 90), positive (*n* = 90), and neutral (*n* = 90) images presented during the working memory and episodic memory tasks. They were presented with a fixation cross for 1 s, an image for 2 s, and then were asked to rate the image in terms of valence and arousal using a 5-point Likert scale.

### Data Analyses

The analyses were conducted in IBM SPSS Statistics for Windows, Version 27.0. We first analyzed the participant’s ratings of the images as a manipulation check to ensure that the participants in our sample rated the images as negative, positive, or neutral as intended. Differences in valence and arousal ratings were analyzed using a one-way ANOVA.

To analyze recognition performance in both the working and episodic memory tasks, we used the drift diffusion model (Ratcliff, 1978; Fig. 1). The fast-dm program (Voss & Voss, 2007) was used to estimate each participant’s parameters of the model and the Maximum likelihood estimation (MLE) approach was chosen given that we had few trials per subject (Myers et al., 2022). If a participant’s data did not converge, we excluded their data. We assessed the goodness-of-fit of the model by running a sanity check, predictive check, and parameter recovery study (Myers et al., 2022). Once the model fit was assessed, we varied the number free parameters (i.e., removing starting point as a free parameter) and compared the model fit using the Akaike’s Information Criterion (AIC; Akaike, 1974). The AIC is used to compare the log-likelihood estimate (LLE) between models, where a smaller AIC indicates a better fit. We used the model with the better fit (i.e., lower AIC) for the working memory task and the episodic memory task, independently. A drift rate for each valence (positive, negative, neutral) and trial type (old, new) was calculated for each participant, that is, six drift rates per task per participant. Although there were high and low load trials in the working memory task, the drift rate was not calculated separately based on load because there were too few trials of each load per valence and trial type.

After estimating the model fit for each participant for each task, the drift rates were analyzed using multivariate multiple regressions for the working memory task. Given the theoretical background, we hypothesized the interference of negative versus positive distractors would disrupt performance for both old and new trials. Thus, the outcome variables were the average drift rate (old drift rate and absolute value of new drift rate) per valence to assess the overall interference of the emotional distractors by valence. Higher values indicate a higher evidence accumulation across items, suggesting less interference, compared with lower values, which would suggest a lower evidence accumulation (i.e., more interference).

For the episodic memory task, the outcome variable, the drift rate for the old positive stimuli, was examined using a linear regression. To assess whether anhedonia (predictor) was associated with the drift rate, the DARS score was used as a predictor while controlling for age, gender, medication use, and depression symptom severity (BDI-II score).

Lastly, whether anhedonia severity is associated with the ability to engage in different cognitive strategies during working memory that may associate with episodic memory performance remains unknown. We conducted an exploratory analysis following Weintraub-Brevda and Chua (2019) to examine to what extent working memory success is associated with engagement of distractor images (i.e., compared with suppressing or ignoring), which leads to enhanced episodic memory for that image. To do so, we first identified images that were presented as distractors in a correct working memory trial and then subsequently accurately recognized during the episodic memory task (“working memory correct, and episodic memory remembered”). Then, we calculated the proportion of “working memory correct and episodic memory remembered” trials out of all trials that were remembered during the episodic memory task per valence. We did to control for the expected unequal valence distribution between the forgotten and remembered images.

We also examined to what extent working memory success is associated with diminished episodic memory for the distractor image. To examine those images that were possibly suppressed or ignored during the working memory task, we calculated the number of images that were distractors for a correct working memory trial but were then forgotten during the episodic memory test (“working memory correct, and episodic memory forgotten”) per valence. The proportion of “working memory correct and episodic memory forgotten” trials out of all trials that were forgotten during the episodic memory task were calculated. We ran two multivariate multiple regressions, one for the remembered proportions and the second for the forgotten proportions described above using the DARS scores as a predictor while controlling for age, gender, medication use, and depression severity (BDI-II score).

## Results

### Descriptives and Preliminary Analyses

Participants (*n* = 108) were primarily college-aged (*M*_*age*_ = 20.1; *SD*_*age*_ = 3.55), non-Hispanic White (58.3%), female (83.3%) participants. Thirty-six (33.3%) participants reported to be taking psychotropic medication (e.g., antidepressants) and 28 (25.9%) participants self-reported a diagnosis of major depressive disorder. Their depression (BDI-II) scores ranged from *none* (*0*) to *severe* (*53*), with an average score of mild depression (*M* = 16.95, *SD* = 12.93). The average score on the SHAPS was a 2.55 (*SD* = 2.87; range 0–12). A score greater than two is on the clinical range for anhedonia (Snaith et al., 1995), indicating that our sample’s average fell slightly above the clinical cutoff for anhedonia. The average score on the DARS was a 47.58 (*SD* = 15.37; range 3–68). Compared with the average DARS score of 36.3 (*SD* = 15.5) in a depressed sample, and 15.1 (*SD* = 8.8) in a treatment-refractory depressed sample (Rizvi et al., 2015), our sample had on average lower anhedonia severity (i.e., greater pleasure, greater effort) than previous samples (see Table 1 for all demographic characteristics).

**Table 1:** Demographic and symptom characteristics

Characteristic	
Age, years, *mean (SD)*	20.1 (3.55)
Gender, *n (%)*	
Female	90 (83.3)
Male	17 (15.7)
Nonbinary	1 (0.9)
Race, *n (%)*	
Alaskan Native/Native American	1 (0.9)
Asian	16 (14.8)
Black	1 (0.9)
Non-Hispanic White	63 (58.3)
Latinx/Hispanic (White)	13 (12)
Latinx/Hispanic (non-White)	4 (3.7)
Multiracial	8 (7.4)
Other	1 (0.9)
Unknown	1 (0.9)
Symptom score,* mean (SD)*	
SHAPS	2.55 (2.87)
BDI-II	16.95 (12.93)
DARS^+^	47.58 (15.37)
Self-reported psychiatric diagnosis,* n (%)*	
Major depressive disorder	28 (25.9)
Generalized anxiety disorder	34 (31.5)
Post-traumatic stress disorder	9 (8.3)
Eating disorder	11 (10.2)
Obsessive–compulsive disorder	8 (7.4)
Autism spectrum disorder	2 (1.9)
Panic disorder	4 (3.7)
Social anxiety disorder	10 (9.3)
Attention-deficit hyperactivity disorder	4 (3.7)
Psychotropic medication use, *n (%)*	36 (33.3)

*N* = 108 ^+^ Higher scores indicate higher reported pleasure

The goal was to compare between groups; however, based on the unimodal distribution of the DARS (skewness =  −0.83) and BDI-II (skewness = 0.69) scores, the data were analyzed on a dimensional scale to better capture individual variability. Given that we planned to use the DARS score as a predictor while controlling for BDI-II, we assessed the correlation. Contrary to what we expected based on prior literature (Rizvi et al., 2015), DARS and our depression measure (i.e., BDI-II) were highly correlated (*r* = −0.74, *p* < 0.001) and would limit our ability to detect significant effects in the multivariate multiple regressions. Therefore, moving forward, we examined anhedonia severity and depressive symptoms severity separately.

Prior to conducting any analyses, outliers and missing data were examined. Three participants completed the first visit but did not return for the second visit; therefore, their data was only included for the working memory analyses. Three additional participants completed both visits, but we had to exclude their working memory task data due to a task error resulting in a total of 105 participants who complete each task. To clean the data, first, we excluded a participant’s task data if they responded to less than 50% of trials (working memory task *n* = 2; episodic memory task *n* = 0) and removed “no response” trials across participants (working memory task: 11.9% trials; episodic memory task: 2.8% trials) to run the drift diffusion model. Next, outlier reaction times (RTs) were calculated using Tukey’s method (Tukey, 1977) of interquartile range (IQR = Q3-Q1) to filter low outliers (Q1-k*IQR) and high outliers (Q3 + k*IQR), using a k-value of 1.5. We excluded participants with more than 10% of total trials as RT outliers (working memory task: *n* = 3; episodic memory task: *n* = 5). Of the remaining 100 participants in the working memory task, a total of 44 trials (0.6%) were excluded as outliers. The final data set for the working memory task consisted of 8,559 trials. Participants with excluded data had similar BDI-II (*M* = 16.75, *SD* = 13.40) and DARS (*M* = 47.00, *SD* = 16.00) scores as the complete sample; that is, they did not exhibit greater depressive or anhedonia symptoms. Similarly of the remaining 100 participants in the episodic memory task, a total of 452 trials (2.6%) were excluded as outliers. The final data set for the episodic memory task consisted of 17,544 trials.

### Manipulation check

We assessed whether the valence and arousal ratings from the study participants aligned with the IAPS-normed categories. A one-way ANOVA yielded a significant main effect of valence on the study valence ratings, *F*(2, 267) = 593.02,* p* < 0.001, η_p_^2^ = 0.82, following the normed pattern. That is, the positive images were rated as more pleasant, followed by the neutral and negative images using a 1–5 scale, *M*_*rating*_ positive = 3.76 [*SD* = 0.32], *M*_*rating*_ neutral = 2.74 [*SD* = 0.48], *M*_*rating*_ negative = 1.75 [*SD* = 0.36]). The arousal ratings also aligned with the normed arousal ratings (*M*_*rating*_ positive = 2.27 [*SD* = 0.34], *M*_*rating*_ neutral = 2.03 [*SD* = 0.32], *M*_*rating*_ negative = 2.69 [*SD* = 0.34]), that is, all in the mid-arousal range for a 1–5 scale (2.33–3.66). Similarly to the normed data, a one-way ANOVA yielded a significant main effect of valence on the arousal ratings, *F*(2, 267) = 90.49,* p* < 0.001, η_p_^2^ = 0.4, where the negative images were rated more exciting, followed by the positive and neutral images (all *p* < 0.001).

Task performance descriptives using the signal detection model (Green & Swets, 1966) are presented in Tables 2 and 3 to facilitate comparison with previous research.

**Table 2:** Signal detection model parameters for the working-memory task

Parameter		*M* (*SD*)
HR_pos	Low	0.73 (0.19)
	High	0.56 (0.25)
HR_neu	Low	0.77 (0.2)
	High	0.61 (0.24)
HR_neg	Low	0.71 (0.2)
	High	0.57 (0.23)
FAR_pos	Low	0.11 (0.16)
	High	0.11 (0.13)
FAR_neu	Low	0.09 (0.11)
	High	0.17 (0.15)
FAR_neg	Low	0.16 (0.16)
	High	0.18 (0.16)
*d*'_pos	Low	1.85 (0.86)
	High	1.31 (0.85)
*d*'_neu	Low	2.01 (0.75)
	High	1.30 (0.87)
*d*'_neg	Low	1.61 (0.85)
	High	1.11 (0.88)
RT_pos (*s*)	Low	1.31 (0.23)
	High	1.39 (0.22)
RT_neu (*s*)	Low	1.30 (0.24)
	High	1.43 (0.2)
RT_neg (*s*)	Low	1.36 (0.23)
	High	1.45 (0.2)

*HR=*hit rate, *FAR=*false alarm rate, *d’*=sensitivity index, *RT=*reaction time, *pos=*positive, *neg=*negative, *neu=*neutral, *low=*low working memory load, *high=*high working memory load

**Table 3:** Signal detection model parameters for the episodic memory task

Parameter	*M* (*SD*)
HR_pos	0.45 (0.02)
HR_neu	0.44 (0.02)
HR_neg	0.66 (0.02)
FAR_pos	0.12 (0.01)
FAR_neu	0.13 (0.01)
FAR_neg	0.16 (0.01)
*d*'_pos	1.22 (0.63)
*d*'_neu	1.14 (0.7)
*d*'_neg	1.59 (0.75)
RT_pos (*s*)	1.00 (0.12)
RT_neu (*s*)	1.00 (0.12)
RT_neg (*s*)	1.04 (0.13)

*HR=*hit rate, *FAR*=false alarm rate, *d’*=sensitivity index, *RT=*reaction time, *pos*=positive, *neg*=negative, *neu=*neutral

### Drift Diffusion Model – Working Memory Task

**Fig. 3:**
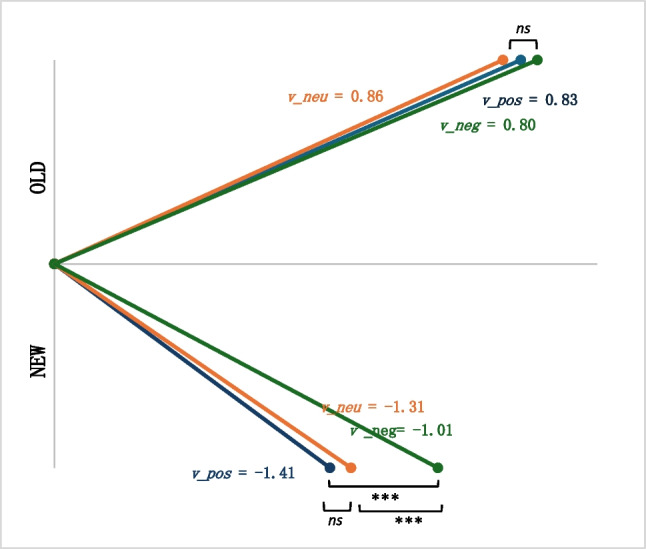
Drift diffusion model drift rate for working-memory task. *v* = drift rate; pos = positive; neg = negative; neu = neutral; ns = not significant; ***p < 0.001

The drift diffusion model converged for 82 participants and failed to converge for 18 participants who had less than ten trials for at least one of the valence x status categories. Therefore, the following analyses were conducted only with the convergent data. To assess model fit, we first visually checked the data to make sure the parameter values were reasonable and appropriate. We ran a predictive check for all participants and ran the construct-samples tool from fast-dm package (Voss & Voss, 2007; Voss et al., 2015) to generate simulated raw data from DDM parameters. We then performed a parameter recovery analysis by running the drift diffusion modeling for the simulated data. The DDM parameters from the empirical data and simulated data were compared and highly correlated (all *r* > 0.87), indicating an appropriate model fit. Lastly, we varied the number of free parameters and compared the AIC values between models. We found that a model where the *starting value*, *nondecisional time*, and *boundary separation* were equal across conditions (i.e., constant within participant), each parameter’s variances fixed at zero, and the *drift rate* varied per valence and trial status (i.e., six drift rates per participant) had better fit compared with models where the *starting value* varied by valence and where the variances varied by participant. The average DDM parameters for the working memory task across participants are presented in Table 4 and Fig. 3. Based on post-hoc one-sample *t*-tests, all drift rates significantly differed from zero, *p* < 0.001. There was no statistically significant valence effect among the old drift rates, *F*(2, 162) = 0.749,* p* = 0.47, η_p_^2^ = 0.01, whereas there was a significant valence difference within the new drift rates, *F*(2, 162) = 31.35,* p* < 0.001, η_p_^2^ = 0.28. The drift rate for trials with new abstract stimuli and negative distractors (*M* = − 1.01, *SD* = 0.66) was significantly lower than those with neutral distractors (*M* = − 1.31, *SD* = 0.63) and positive distractors (*M* = − 1.41, *SD* = 0.7; all *p* < 0.001).

**Table 4:** Working-memory task –drift diffusion model parameters

Parameter	*M* (*SD*)
zr	0.51 (0.07)
a	1.99 (0.38)
t_0_	0.62 (0.15)
v_pos_old	0.83 (0.66)
v_neu_old	0.86 (0.66)
v_neg_old	0.80 (0.65)
v_pos_new	− 1.41 (0.7)
v_neu_new	− 1.31 (0.63)
v_neg_new	− 1.01 (0.66)

*zr=*starting point, *a=*boundary separation, *t*_*0*_=nondecisional time, *v=*drift rate, *pos=*positive, *neg=*negative, *neu=*neutral, *new=*new trials, *old=*old trials

**Fig. 4:**
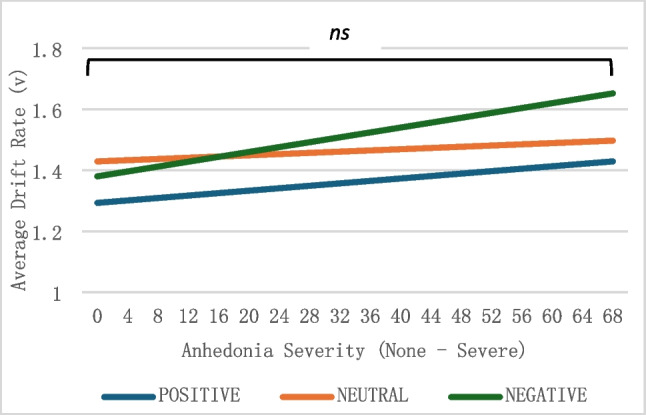
Average drift rate for working memory task. DARS scores were recoded for visual representation. High scores indicate more anhedonia symptoms. *ns* = not significant

We hypothesized that as anhedonia severity increased, the average drift rate of old and new trials with negative distractors in the working memory task would decrease, and the average drift rate of old and new trials with positive distractors in the working memory task would increase. Contrary to our hypotheses, based on a multivariate multiple regression, score on the DARS did not statistically significantly predict the average drift rates of negative, positive, or neutral trials, *F*(3, 75) = 0.61,* p* = 0.61, η_p_^2^ = 0.02 (Fig. 4), when controlling for age, gender, and psychiatric medication use. That is, anhedonia severity was not associated with the rate of accumulation for neutral abstract shape stimuli when the distractors were emotionally valenced.

As a post-hoc analysis, we assessed whether depression-only was associated with the average drift rates; however, contrary to expectations, BDI-II scores did not predict the drift rates *F*(3, 75) = 1.66,* p* = 0.18, η_p_^2^ = 0.06.

## Drift Diffusion Model – Episodic Memory Task

**Fig. 5:**
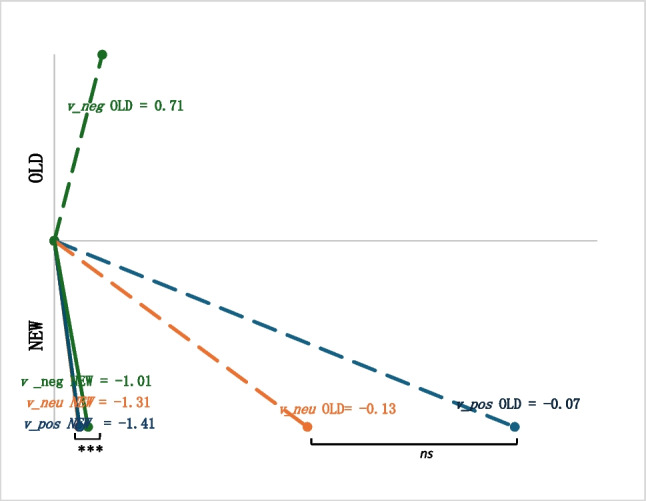
Drift diffusion model for episodic memory task. *v* = drift rate; pos = positive; neg = negative, neu = neutral; ns = not significant; ***p < 0.001; v_neu OLD and v_pos OLD were not statistically different than zero

The drift diffusion model converged for 100 participants. We followed the same procedure as the working memory task to assess model fit. The predictive check and parameter recovery analysis were examined for all participants and similarly yielded an appropriate model fit by comparing the DDM values between the simulated data and the empirical data (*r* > 0.85). The best model was also a model where the *starting value*, *nondecisional time*, and *boundary separation* were equal across conditions (i.e., constant within participant), each parameter’s variances fixed at zero and the *drift rate* varied per valence and trial status (i.e., six drift rates per participant). The average DDM parameters for the episodic memory task across participants are presented in Table 5 and Fig. 5. Based on post-hoc one-sample *t*-tests, the drift rates for the new probes (i.e., lures) significantly differed from zero, *p* < 0.001 across the three valence categories. The drift rates for the old probes (i.e., target images) were statistically different from zero for the negative images, *p* < *0.0*01, but not for the neutral, *p* = 0.06 or positive, *p* = 0.28 images. There was a significant valence effect among the old drift rates, *F*(2, 198) = 173.22,* p* < 0.001, where negative distractors negative target images had larger positive values a quicker accumulation toward an old response (*M* = 0.71, *SD* = 0.65), compared with neutral and positive target images that had lower drift rates with negative values, indicating a response at the new boundary (*M* = 0.13, *SD* = 0.7,* M* = − 0.07, *SD* = 0.68, respectively; all *p* < 0.001). Similarly, there was significant valence effect among the new drift rates, *F*(2, 198) = 51.17,* p* < 0.001, where negative lures had a slower accumulation toward new response (*M* = − 1.01, *SD* = 0.51), compared with neutral and positive lures that had higher drift rates (*M* = − 1.34, *SD* = 0.62, *M* = − 1.38, *SD* = 0.54, respectively; all *p* < 0.001).

**Table 5:** Episodic memory task—drift diffusion model parameters

Parameter	*M* (*SD*)
zr	0.5 (0.05)
a	1.56 (0.21)
t_0_	0.53 (0.12)
v_pos_old	− 0.07 (0.68)
v_neu_old	− 0.13 (0.7)
v_neg_old	0.71 (0.65)
v_pos_new	− 1.38 (0.54)
v_neu_new	− 1.34 (0.62)
v_neg_new	− 1.01 (0.51)

*zr *starting point, *a *boundary separation, *t*_*0*_ nondecisional time, *v *drift rate, *pos *positive, *neg *negative, *neu *neutral, *new *new trials, *old *old trials

**Fig. 6:**
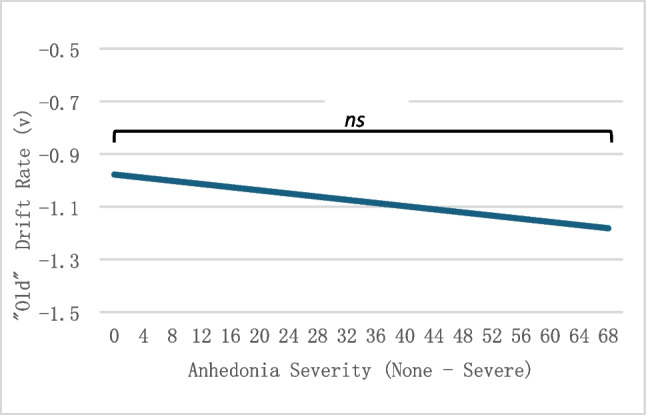
Old drift rate for positive stimuli in the episodic-memory task. DARS scores were recoded for visual representation. High scores indicate more anhedonia symptoms. *ns* = not significant

We hypothesized that as anhedonia severity increased, the drift for positive stimuli for “old” trials (i.e., positive target images) in the episodic memory task would decrease, indicating a lower accumulation of positive stimuli evidence. Similarly to the working memory task, the linear regression results indicated that DARS scores did not statistically significantly predict the “old” positive drift rate, or rate of accumulation of positive target images, *F*(1, 95) = 0.4,* p* = 0.53, η_p_^2^ = 0.004 (Fig. 6).

To assess whether previous depression findings replicated in our sample, we examined whether depression severity predicted the “old” drift rates in a multivariate multiple regression. Although not statistically significant, the findings suggest a trend towards significance where BDI-II trended to predict the “old” drift rates multivariate outcome, *F*(3, 93) = 2.3,* p* = 0.08, η_p_^2^ = 0.07. Given the nonstatistically significant model, the individual regression coefficients were not interpretable.

### Exploratory Analyses – Working and Episodic Memory

We conducted two exploratory multivariate multiple regressions to examine whether the relationship between a successful working memory task and recognition performance was associated with anhedonia. Based on a multivariate multiple regression, anhedonia severity did not predict the proportion of distractors from successful working memory trials that were recognized in the episodic memory task over all the remembered trials, *F*(3, 93) = 2.08,* p* = 0.11, η_p_^2^ = 0.06, while controlling for age, gender, and psychiatric medication use. Similarly, anhedonia severity did not predict the proportion of distractors from successful working memory trials that were forgotten over all the forgotten trials, *F*(3, 92) = 0.88,* p* = 0.45, η_p_^2^ = 0.03.

## Discussion

Although prior literature examining memory performance in depression supports differential episodic memory performance for emotionally valenced stimuli, the mechanism of poor recognition of positive stimuli remains unclear. Similarly, the mechanism of the differential effects of emotionally valenced distractors (i.e., greater interference of negative stimuli and decreased interference of positive stimuli) in working memory processes in depression is inconclusive. The current study was designed to investigate further whether the distinct memory processes were associated with anhedonia symptomology. In addition, we examined working and episodic memory processes using computational modeling to compare the strength of memory retrieval through an index called drift rate. Based on previous literature, we hypothesized that as anhedonia symptomology increased, negative distractors would lead to greater interference in a working memory task, and positive distractors would lead to less interference while retaining an abstract stimulus in mind.

Unexpectedly, the drift rate of evidence accumulation for neutral abstract shapes was not predicted by levels of anhedonia. Although this was the first study to use DDM in a working memory task for anhedonia, we also found that depression alone did not predict the average drift rate. That is, depression severity did not predict differences in the working memory retrieval process when the distractors were negative, neutral, or positive. One potential reason we did not find evidence for this effect comes from a study by Rutherford et al. (2023). The authors demonstrate that the connection between anhedonia symptomology and dysfunction in working memory is a downstream effect of the effect of rumination on working memory. Even though individuals in our sample had varying levels of anhedonia, we did not directly measure rumination severity as suggested in Rutherford study, which was published after our study initiation. Furthermore, we predicted that negative stimuli would more likely cause greater disruption in the maintenance of neutral abstract stimuli in working memory in depression, because it is more likely to be congruent with ruminative thoughts; however, this effect may be greater when the negative stimuli are self-relevant, not general as it was in the current study.

Given prior neuroscientific evidence, we also expected that symptoms of anhedonia would be associated with the efficiency of evidence accumulation of memory for positive stimuli in an episodic memory recognition task. This is the first study to our knowledge to examine the relation between anhedonia severity and memory for positive stimuli. Contrary to our hypotheses, our results did not provide evidence of a relationship between anhedonia and the strength of long-term memory retrieval of positive stimuli using a delayed, incidental recognition memory paradigm. In other words, the rate at which evidence accumulated toward an “old” decision did not vary as anhedonia symptoms increased. One potential reason for the lack of association is that the positive stimuli in our study may not have elicited positive prediction errors (PPEs). While there is no direct method in our study to compare the PPEs generated during the encoding process, the element of surprise is an important component in generating PPEs, such as in the oddball tasks used in previous studies (Maksmimovskiy et al., 2022). Our hypotheses stemmed from prior work demonstrating that dopamine neurons elicit PPEs (Schultz, 2016), and PPEs strengthen memory formation in healthy controls (Jang et al., 2019). We hypothesized that differences in behavioral data would be downstream effects of dampened PPEs in depression due to anhedonia-related dysregulation in the dopamine system (Dillon, 2015). However, if the task did not successfully elicit PPEs, it would not be possible to detect blunted effects in those with greater anhedonia and depressive symptoms. Another possibility for the lack of association is that dopamine dysregulation may not be present in all individuals with depression. For example, Rutledge et al. (2017), using functional neuroimaging, computational modeling and large-scale data found that reward prediction errors were not correlated with depression in a nonlearning context.

Prior studies have found relations between depressive symptoms and drift rates of emotionally valenced stimuli; however, our findings did not replicate the depression findings, although they were statistically trending. The primary differences between our study and Cataldo et al. (2023) and Maksimovskiy et al. (2022) were methodological. First, our sample consisted of primarily college-age participants (*M*_age_ = 20.1; *SD*_age_ = 3.55) with mild depression symptoms (*M*_BDI-II_ = 16.95; *SD*_BDI-II_ = 12.93). While Maksimovskiy’s (2022) participants were also recruited through an undergraduate participant pool, they were slightly older (*M*_age_ = 23.69, *SD*_age_ = 8.91 and *M*_age_ = 23.69, *SD*_age_ = 6.27) and consisted of extreme groups: a low depression group (*M*_BDI-II_ = 2.3, *SD*_BDI-II_ = 1.88) and high depression group (*M*_BDI-II_ = 29.92, *SD*_BDI-II_ = 6.55). Cataldo et al. (2023) recruited through an online platform and was extremely well-powered with a sample size of more than 1,300 participants who were also on average older (*M*_age_ = 32.14, *SD*_age_ = 13.6) than our sample. Second, both prior studies used the Hierarchical Drift Diffusion Model (HDDM) instead of the DDM with the MLE used in the current study. The HDDM is a Bayesian DDM implementation, which generates a posterior distribution for each parameter instead of a best-fitting value, such as the DDM, and creates group-level parameter estimates. Therefore, their more extensive modeling techniques may generate better model parameters. Instead of employing the HDDM, we opted to continue using the DDM with the MLE given that the MLE approach is appropriate to answer our research questions, and it is more accessible to understand and interpret. Lastly, the task paradigms were different. Maksimovskiy et al. (2022) used an oddball paradigm where the positive pictures were shown less frequently than negative and neutral pictures, because they were meant to be surprise positive stimuli and elicit prediction error. Cataldo et al. (2023) used emotionally valenced words as the stimuli. The current study was designed, and data collection began prior to the previous manuscripts were published. Therefore, this study was not designed to replicate their findings with the same behavioral tasks.

As an exploratory analysis, we also examined the relation between working memory and episodic memory performance. Specifically, we followed Weintraub-Brevda and Chua (2019) to examine whether the association of the engagement or disengagement with distractors during the working memory task and recognition performance was different across levels of anhedonia severity. Our findings suggest that participants, across levels of anhedonia, engaged similarly with the distractors on successful working memory trials. That is, there was no difference in their ability to maintain abstract neutral stimuli in working memory by engaging with the distractor (e.g., reappraisal), which was inferred as better episodic memory, or by suppressing or ignoring the distractor, which was deduced as worse episodic memory. Therefore, the results of this study suggest no unique association of anhedonia and the link between working memory and episodic memory performance.

Additional methodological components may have contributed to the unexpected results across tasks. While the IAPS image valance ratings were statistically different based on the normed valence categories, the IAPS image arousal ratings were also statistically different. Given previous literature on the distinct processes for valence and arousal on emotional memory (Kensinger & Corkin, 2004), the valenced stimuli in our study were confounded by arousal levels. As an attempt to match arousal levels post hoc, we manually eliminated arousal extremes to have averages across valences that were not significantly different, resulting in 83 images being eliminated across both tasks (30.74%). The reduced number of stimuli was too small to re-run the analyses with the matched-arousal levels post hoc. Although we did not find significant differences in anhedonia and the drift rates based on valence, in future studies, it is imperative to match arousal levels across valences to ensure that any differences are due to valence and not arousal.

The measurement of anhedonia severity is also an important consideration. Anhedonia may be defined based on the dysfunction of reward processes and on an affective dimensional scale (De Fruyt et al., 2020); however, specific anhedonia symptomology may be associated with poor recognition and differential effects of valenced emotional distractors in working memory tasks. Based on Sherdell et al. (2012), participants with depression had lower anticipatory pleasure during a cartoon-watching task than healthy controls while consummatory pleasure remained comparable. Whereas a meta-analysis by Keren et al. (2018) found that during reward tasks, participants with depression show neural deviations during feedback (i.e., consummatory) and less so during reward anticipation. The differences across findings illuminate the need of further examination on the multiple facets of anhedonia in depression using adequate methodology (Rizvi et al., 2016) and to what extent the distinct anhedonia dimensions associate with memory processes and memory deficits.

Our task was not designed to have the positive stimuli be surprising or less frequent than negative or neutral stimuli, but given previous work involving prediction errors, future studies could account for an element of surprise. In addition, our working memory task included trials with low load (i.e., one abstract shape to remember) and high load (i.e., two abstract shape to remember), but we did not have a sufficient number of trials to use this modeling technique and examine drift rates by load and valence. It is possible that valenced distractors interfere to a different degree in low vs. high load trials.

Future studies can expand our current findings by examining additional cognitive processes using computational modeling to study the mechanisms of the differential memory processes. It will be interesting for future studies to examine if response or memory bias differs in the recognition performance based on anhedonia severity. Furthermore, memory may be assessed through a variety of paradigms, such as free recall, that capture different memory pathways, which can be examined in future studies. In addition, we are using behavioral data to make inferences about the downstream effects of PPEs at encoding. However, future studies can include neurophysiological measures (e.g., electroencephalogram) as a more closely related measure of the encoding process (Polich, 2007). Lastly, future studies can set higher clinical thresholds, include clinician-rated measure of anhedonia, examine whether the association is specific to one facet of anhedonia (e.g., anticipatory), and examine the memory processes in older and more racial-ethnic diverse sample.

The current study found that anhedonia severity and memory retrieval strength for neutral shapes after viewing emotionally valenced distractors were not associated. Similarly, the severity of anhedonia and memory accumulation efficiency were unrelated when recognizing positive stimuli. These findings expand the characterization of the memory processes in depressed young adults, suggesting that the mechanisms underlying self-reported anhedonia symptoms are separate from those underlying the studied memory processes. Therefore, the current study indicates that individuals with depression and higher anhedonia symptoms are equally as likely to experience differential memory processes as individuals with depression and lower anhedonia symptoms. Understanding memory processes in depression remains crucial to inform interventions and also to target maintaining factors effectively.

## Supplementary Information

Below is the link to the electronic supplementary material.

Supplementary file1 (DOCX 28 KB)

## Data Availability

The data and materials for all experiments are available upon request. All procedures were preregistered (https://doi.org/10.17605/OSF.IO/Q7GA8).

## Declarations

### Open practices statements

The data and materials for all experiments are available upon request. All procedures were preregistered (https://doi.org/10.17605/OSF.IO/Q7GA8).
