# Supplementary material for: Examining working and episodic memory in young adults with anhedonia
Source: Cogn Affect Behav Neurosci. 2025 Jun 2;25(6):1807–21. doi: 10.3758/s13415-025-01315-y (PMC12615527; doi:10.3758/s13415-025-01315-y)
Supplement: Supplementary file 1 — Supplementary file1 (DOCX 28 KB) [file 13415_2025_1315_MOESM1_ESM.docx]

**Supplementary material**

The supplementary material consists of the results from using the correct recognition score (hits – correct rejections) and sensitivity parameter (d prime) instead of the drift rates as the outcome measure.

**Working Memory Task**

Based on a multivariate multiple regression, score on the DARS did not statistically significantly predict the corrected recognition score of working memory trials with negative, positive, or neutral trials, *F*(3, 93)=1.22, *p*=.31, ηp^2^ = .04, nor the d’ value, *F*(3, 93)=1.45, *p*=.23, ηp^2^ = .05, when controlling for age, gender, and psychiatric medication use.

**Episodic Memory Task**

Similarly, for the episodic memory task, DARS scores did not statistically significantly predict the corrected recognition score of negative, positive, or neutral images, *F*(3, 93)=1.14, *p*=.34, ηp^2^ = .04, nor the d’ value, *F*(3, 93)=1.24, *p*=.30, ηp^2^ = .04, when controlling for age, gender, and psychiatric medication use.
